# Bacteria from foods and gut microbiota produce methylglyoxal and this metabolite leads to the formation of bioactive 1-acetyl-β-carboline alkaloids

**DOI:** 10.1038/s41598-026-35162-9

**Published:** 2026-01-09

**Authors:** Tomás Herraiz, Ana Sánchez-Arroyo, Blanca de las Rivas, José María Landete, Rosario Muñoz

**Affiliations:** 1 Instituto de Ciencia y Tecnología de Alimentos y Nutrición, ICTAN-CSIC, C/José Antonio Nováis 6, Madrid, Spain; 2 Instituto Nacional de Investigación y Tecnología Agraria y Alimentaria, INIA-CSIC, Carretera de La Coruña Km 7.5, Madrid, Spain

**Keywords:** Methylglyoxal, 1-acetyl-β-carboline, methylglyoxal synthase (MgsA), *Lactobacillaceae*, *E. coli*, *L. sakei*, Gut microbiota, Foods, Biochemistry, Biotechnology, Microbiology

## Abstract

Methylglyoxal (MGO) is a highly reactive and toxic compound whereas 1-acetyl-β-carbolines (ACE-βCs) are bioactive alkaloids. These compounds were studied in cultures of bacteria from foods and human gut microbiota*.* Two ACE-βCs were identified as 1-acetyl-β-carboline (AβC) and 1-acetyl-β-carboline-3-carboxylic acid (AβC-COOH). Cultures containing ACE-βCs also contained MGO that was produced during bacterial growth. MGO produced by bacteria reacted spontaneously with L-tryptophan (L-Trp) and afforded ACE-βCs. MGO and ACE-βCs appeared in cultures from *Escherichia coli* and *Lactilactobacillus sakei* but not in cultures from other lactobacilli (*L. plantarum*, *L. rhamnosus*, *and L. paracasei*). Those bacteria producing MGO contained the enzyme methylglyoxal synthase (MgsA). MgsA was needed for the production of MGO as demonstrated by expressing the *mgsA* gene from *L. sakei* DSM 15831^ T^ into *Lacticaseibacillus paracasei* BL23, and the recombinant strain produced MGO. The factors involved in the bacterial production of MGO are highlighted. *E. coli* produced MGO only in presence of glucose and *L. sakei* on galactose. The bacterial production of MGO (and resultant ACE-βCs) increased with the concentration of carbohydrates (glucose or galactose). The production of MGO from glucose in *E. coli* highly increased when phosphate was added and higher levels were produced under anaerobic or oxygen-limited conditions than in aerobic conditions. The results suggest that *E coli* may produce MGO under gut conditions and MGO may result from the accumulation of phosphorylated intermediates in glycolysis. It is concluded that bacteria possessing MgsA present in foods and human gut microbiota produce MGO that leads to the formation of ACE-βCs alkaloids. Bacterial production of MGO is relevant owing to its reactive and toxic nature whereas ACE-βCs are bioactive substances investigated in different targets.

**Supplementary Information:**

The online version contains supplementary material available at 10.1038/s41598-026-35162-9.

## Introduction

Methylglyoxal (MGO) is an α-dicarbonyl compound generated during cellular metabolism that has attracted much attention for decades owing to its biological implications. Although the physiological levels of MGO are generally low, it could play a role in cellular signaling while elevated levels have been implicated in various pathological conditions, including diabetes, neurodegenerative diseases, and cardiovascular disorders^1–5^. In fact, MGO is a potent electrophile that modifies proteins and DNA and forms advanced glycation end products (AGEs) that may lead to cellular dysfunction and tissue damage, contributing to the pathogenesis of various diseases^4,6^. Cells possess mechanisms to detoxify MGO, such as the glyoxalase system and aldo–keto reductases^3,7,8^. Owing to these implications, additional research is needed on the metabolism of MGO and its physiological and pathological roles.

MGO is primarily formed in vivo as a by-product of glycolysis through the non-enzymatic degradation of triose phosphates or alternative pathways such as the degradation breakdown of lipids or threonine^9^. However, in certain microorganisms MGO can be produced enzymatically through the specific action of the enzyme methylglyoxal synthase (MgsA). This enzyme catalyzes the conversion of dihydroxyacetone phosphate (DHAP), an intermediate in glycolysis, into MGO and inorganic phosphate and plays a significant role in bacterial metabolism^10^. This pathway is known as the methylglyoxal bypass and it has physiological significance as an alternative metabolic pathway in the metabolism of carbohydrates during glycolysis in some bacteria like *E. coli*^11,12^*.* The presence of MgsA in bacteria may play a role in their adaptation to stress conditions including nutrient limitations^11,12^. MGO may affect the biology of bacteria by impacting their growth, survival, and interaction with the environment^11^. Currently, the production of MGO by bacterial species present in foods and the human gut along with its impact on the host are emerging areas of research^13,14^.

β-Carbolines (βCs) are a family of naturally-occurring bioactive alkaloids which have antimicrobial, antiparasitic and anticancer activities among others^15–20^. Among them, 1-acetyl-β-carbolines (ACE-βCs) occur in foods as a result of the reaction of L-tryptophan (L-Trp) with MGO derived from carbohydrate degradation^21,22^. These β-carbolines may be also formed under physiological conditions and in human serum^22^. ACE-βCs are inhibitors of key kinase enzymes, have anti-filamentation and anti-inflammatory activities and are being investigated in other targets^23,24^. Recently, the presence of the bioactive 1-acetyl-β-carboline (AβC) was reported in lactobacilli cultures and assumed that it was produced by those baceria^23^; however this production was not confirmed later^21^. Nevertheless, it was hypothesized that microorganisms with MgsA could synthetize MGO, which eventually could led to formation of AβC^21^. Now, results reported in this work contribute to settle this issue.

MGO plays a significant role in the biology of bacteria by impacting their growth, survival, and interaction with host along with contribution to virulence^11.^ Therefore, unraveling the production of MGO by bacterial species present in foods and human gut microbiota is an interesting topic that could help to understand the biological implications of this toxic metabolite. This work studies the formation of MGO in bacterial cultures from *E. coli* and lactobacilli and describes the main factors involved, including the presence of carbohydrates, phosphate and different aeration conditions. In addition, it describes the presence of ACE-βCs in the cultures from those bacteria producing MGO. The results obtained offer new clues on the biosynthesis of MGO by bacteria present in foods and human gut microbiota and on the factors involved as well as on the formation of the bioactive ACE-βCs alkaloids. The potential biological implications of these results for the bacterial metabolism and human health are discussed.

## Material and methods

### Materials, strains and growth conditions.

The lactic acid bacteria (LAB) strains used in this study were *Lactilactobacillus sakei* subsp*. carnosus* DSM 15831^ T^ (genome accession number GCA_001435715.1), *Lactilactobacillus sakei* subsp. *sakei* DSM 20017^ T^ (GCA_002370355.1), *Lacticaseibacillus rhamnosus* GG (GCA_003353455.1), *Lactiplantibacillus plantarum* WCFS1 (GCA_000203855.3), *Lacticaseibacillus paracasei* BL23 (GCA_000026485.1), and *Lactococcus cremoris* subsp. *cremoris* MG1363 (GCA_000009425.1). *L. plantarum* WCFS1 strain was kindly provided by Dr. Michael Kleerebezem (NIZO Food Research, The Netherlands). LAB strains were routinely grown at 30 °C in MRS broth (Pronadisa, Spain) under aerobic conditions, except *L. cremoris* MG1363 which was grown at 30 °C in M17 broth supplemented with 0.5% glucose. Recombinant LAB strains harbouring pNZ:TuB.MgsA were grown in their corresponding media containing chloramphenicol (5 μg/mL). *Escherichia coli* BL21(DE3) (Invitrogen) was cultured in Luria–Bertani (LB) medium at 37 °C and shaking at 140 rpm. Table S1 shows the bacterial strains used in this study from species frequently isolated from foods and gut microbiota and some of them possessing MgsA. The compounds methylglyoxal (MGO) and L-tryptophan (L-Trp) were obtained from Merck. The ACE-βCs compounds: 1-acetyl-β-carboline (AβC) and 1-acetyl-β-carboline-3-carboxylic acid (AβC-COOH) were previously synthetized and chemically characterized^22^.

In the studies aimed at determining the effects of several compounds on the production of MGO and ACE-βCs, a modified MRS medium (mMRS) was used, in which the presence of dipotassium phosphate (2 g/L) and glucose (20 g/L) was omitted. Therefore, the composition of the mMRS medium was the following: casein peptone (tryptic digest) (10 g/L), meat extract (10 g/L), yeast extract (10 g/L), Tween 80 (1 g/L), sodium acetate (5 g/L), ammonium citrate (2 g/L), magnesium sulphate (0.2 g/L), and manganese sulphate (0.2 g/L) and the pH was adjusted to 6.5. The mMRS media was sterilized by autoclave. For the different assays performed on this study, sterilized mMRS was supplemented with glucose (from 0 to 20%), galactose (from 0 to 20%), dipotassium phosphate (from 0 to 20 g/L) or L-Trp (at 0.6 mg/mL) with recently prepared stock solutions of each compound that were filter-sterilized. The different mMRS media (10 mL into 20 mL glass tubes) were inoculated with bacteria previously grown in their corresponding culture media and incubated at 37 °C in oxygen-limited conditions. Lactic acid bacteria were grown in static cultures whereas *E. coli* cultures were routinely grown under agitation (140 rpm) in an incubator shaker. *E. coli* BL21(DE3) cultures were also incubated under increased aeration conditions (10 mL into 100 mL-Erlenmeyer flask at 140 rpm in an incubator shaker) and strict anaerobic conditions (10 mL into 20 mL glass tubes under static conditions in sealed jar using GasPak™ EZ Anaerobe Container System Sachets (BD, USA)). For each experiment, cultures were carried out at least in duplicate and generally in triplicate. Samples were collected for pH and OD_600nm_ determinations at 0, 24, 48, 72 h and 144 h, and were centrifuged at 12,000 × g, and the supernatants were immediately frozen (− 80 °C). Subsequently, those samples were thawed and analyzed for MGO and ACE-βCs by HPLC and HPLC–MS. Incubated media without cells were used as controls.

### Cloning of *L. sakei mgsA* gene in lactic acid bacteria

The gene encoding MgsA from *Latilactobacillus sakei* subsp. *carnosus* DSM 15831^ T^ (accession WP_056948772.1) was amplified by PCR using Fw-(5′- CCATGGAAATTGCATTAATTGCACACG) and Rv (-5′ TTTTCTAGATTATAGATTGATTAGATTAGAATCTTG) oligonucleotides. The PCR product was digested with *Nco*I and *Xba*I restriction enzymes and ligated into vector pNZ:TuB^25^ digested with the same enzymes. Restriction enzymes and T4 DNA Ligase came from Biolabs (New England Biolabs, Hitchin, UK). The ligation mixture harbouring pNZ:TuB.MgsA was initially transformed into *L. cremoris* MG1363 by electroporation and transformants were selected with chloramphenicol (5 µg/mL, Sigma Aldrich) and confirmed by PCR^26.^ Transformants containing the recombinant pNZ:TuB.MgsA plasmid were checked by PCR by using a primer designed on the vector (forward, For-pNZ 5´-GGAATTGTCAGATAGGCCTAATGACTGG) and the reverse primer (Rv) used to amplify the *L. sakei mgsA* gene. Later, pNZ:TuB.MgsA plasmid was transformed into *L. paracasei* BL23 as described before^26^ and *L. paracasei* BL23 (pNZ:TuB.MgsA) transformants were selected in MRS media containing chloramphenicol (5 µg/mL, Sigma Aldrich) and confirmed by PCR (Figure S1).

### Analysis of MGO in the bacterial cultures.

Bacterial culture supernatants were centrifuged and derivatized with *o*-phenylenediamine (*o*-PDA) to determine MGO. Briefly, 100 μL of culture supernatants were added with 100 μL *o*-PDA solution (conc. 2 mg/mL) and 300 μL of phosphate buffer 0.4 M pH 9.2 and incubated at 40 ºC for 4 h in the dark; then 200 μL of acetonitrile (ACN) was added and MGO analyzed by HPLC as mentioned below with absorbance detection at 317 nm^22^.

### Analysis of MGO and ACE-βCs (AβC-COOH and AβC) by HPLC and HPLC–MS.

The supernatants of microbial cultures were centrifuged and analyzed for ACE-βCs and MGO after its derivatization. The chromatographic analysis was performed with a 1050 high performance liquid chromatograph (Agilent Technologies) coupled to 1100 series DAD. The separation was carried out with a 150 mm x 3.9 mm, 5µm, Novapak C18 column (Waters). The eluents were 50 mM ammonium phosphate buffer adjusted to pH 3 with 85% phosphoric acid (Eluent A) and 20% of eluent A in ACN (Eluent B). The gradient was set from 0 to 32%B in 8 min, then 90%B at 12 min. The flow rate was 1 mL/min, oven temperature was 40 ºC, and the injection volume was 20 µL. The analysis of MGO was determined following derivatization with *o*-PDA to give 2-methylquinoxaline and detected by absorbance at 317 nm. A calibration curve was constructed with MGO standard. The analysis of ACE-βCs was done by injecting the supernatant and the detection of AβC and AβC-COOH was carried out with absorbance at 375 and 280 nm. The concentration of AβC and AβC-COOH was obtained with a calibration curve of synthetized standards analyzed by HPLC as indicated above with detection at 280 nm.

The identification of compounds was accomplished by HPLC-MS by using a Waters Alliance e2695 Separations Module coupled to a quadrupole Waters Acquity QDa mass spectrometer working under positive electrospray ionization (ESI+)^21^. Separation was accomplished with a 2.1 x 100 mm, 3 µm, 100Å, C18 Atlantis T3 column (Waters) and chromatographic conditions were as previously reported^21^. In addition, identification of ACE-βCs was accomplished by using an Agilent HPLC-MS apparatus with an HPLC 1200 series pump coupled to a 6110 quadrupole-MS spectrometer (Agilent)^22^. A Zorbax SB C18 2.1 x 150 mm column (Agilent) was used for separation with eluents A: 0.1 formic acid and B: 20% A in ACN and a program from 0 to 32 % B in 8 min and at 12 min 90% B that was kept until 15 min. The flow rate was 0.6 mL/min, the column temperature was 40 ºC and the injection volume was 20 μL. The MS worked under ESI+ with capillary voltage 3000 V, drying gas of 12.5 L/min, drying gas temperature of 350 ºC, quadrupole temperature of 100 ºC and fragmentation voltage of 90 V. Under these conditions, AβC-COOH and AβC eluted at 11.6 and 12.3 min and gave the ions 255 [M+H]^+^ and 211 [M+H]^+^ respectively.

### Statistical analysis

Data were analyzed for statistical significance with the Student´s t-test and one-way ANOVA with post hoc test applied for multiple comparison.

## Results

### ACE-βCs and MGO in bacterial cultures.

**Fig. 1:**
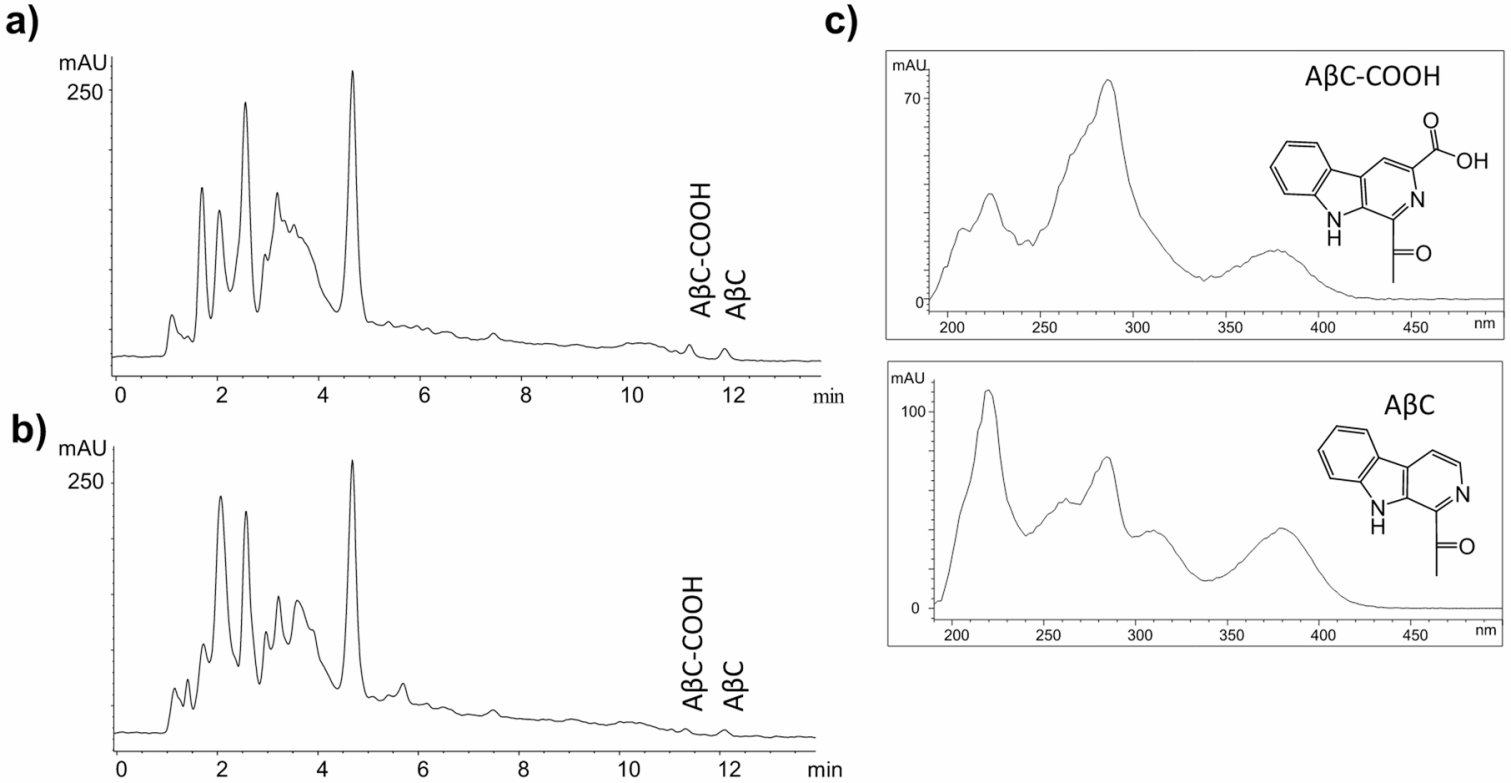
HPLC chromatograms (detection at 280 nm) of culture supernatants (144 h) from *E. coli* BL21(DE3) grown in mMRS medium containing glucose (2%) (**a**) and *L. sakei* subsp. *carnosus* DSM 15831^ T^ grown in mMRS medium containing galactose (2%) (**b**). UV–VIS spectra of the ACE-βCs peaks (AβC-COOH and AβC) (**c**).

**Fig. 2:**
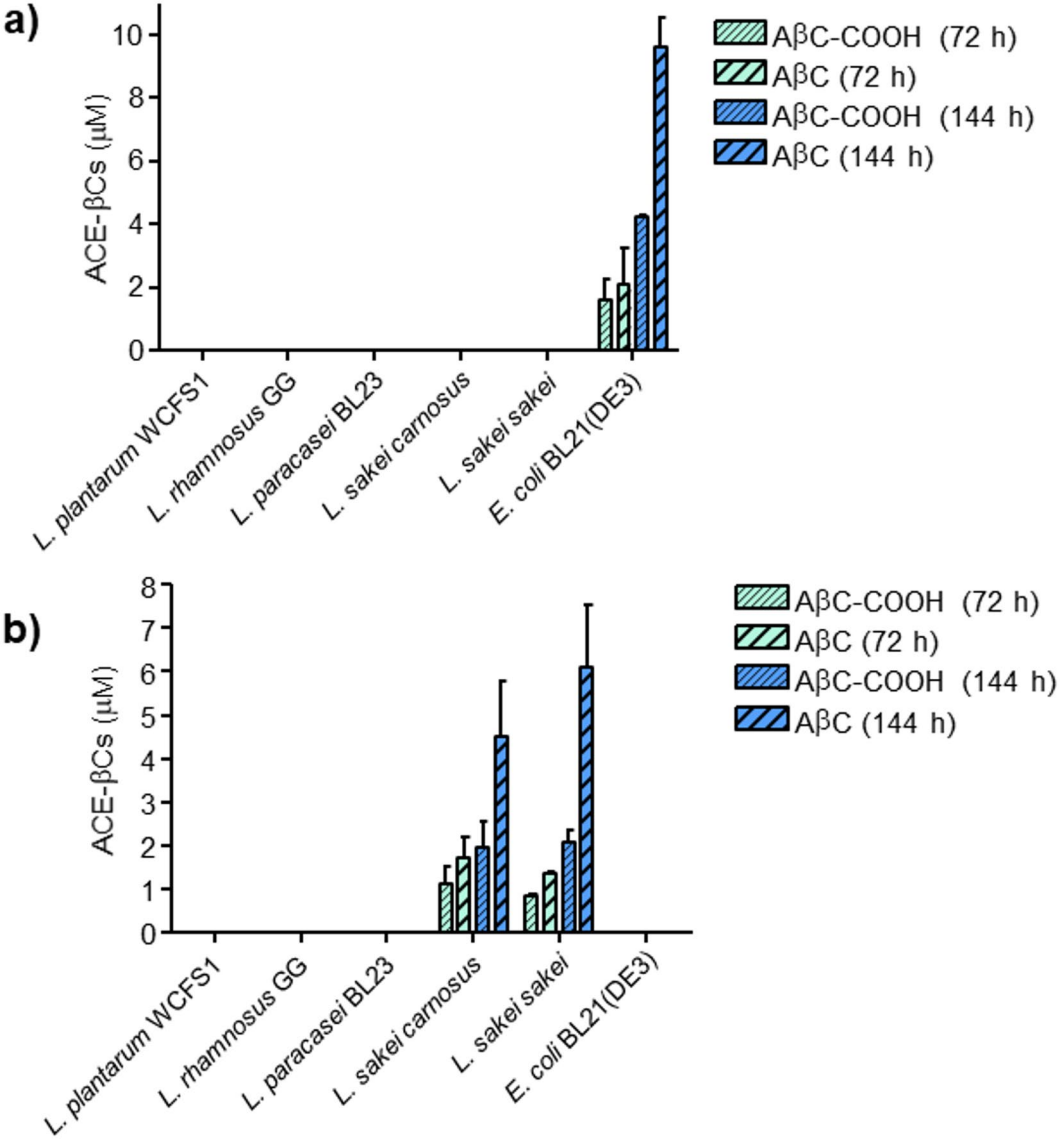
Presence of ACE-βCs (μM) in supernatants of bacterial cultures grown in mMRS media containing glucose (2%) (**a**) or galactose (2%) (**b**). ACE-βCs were analyzed after 72 h and 144 h of incubation.

It has been hypothesized that bacteria present in foods or human gut microbiota might produce methylglyoxal (MGO) which could led to the formation of bioactive 1-acetyl-β-carboline alkaloids (ACE-βCs)^21,22.^ This hypothesis was addressed here by assessing the presence and formation of ACE-βCs in cultures of lactic acid bacteria (*L. plantarum* WCFS1, *L. sakei* subsp*. carnosus* DSM 15831^ T^, *L. sakei* subsp. *sakei* DSM 20017^ T^, *L. rhamnosus* GG, and *L. paracasei* BL23) and *E. coli* BL21(DE3) grown in mMRS media supplemented with two different carbon sources: glucose and galactose as a non-repressive carbon source. Among the seven bacteria assayed, two ACE-βCs were detected in the culture supernatants of *E. coli* and *L. sakei* strains and subsequently identified by chromatographic and spectroscopic data^21,22^ as 1-acetyl-β-carboline-3-carboxylic acid (AβC-COOH) and 1-acetyl-β-carboline (AβC) (Figs. 1 and S2). Quantitative analysis of these ACE-βCs by HPLC–DAD indicated that they formed with time, and prolonged incubation (e.g.144 h vs 72 h) showed higher levels of ACE-βCs in the supernatants of *E. coli* and *L. sakei* (Fig. 2). In contrast, the culture supernatants of the other lactobacilli assayed (*L. plantarum* WCFS1, *L. rhamnosus* GG, and *L. paracasei* BL23) did not contain appreciable amounts of ACE-βCs (Fig. 2). ACE-βCs were detected in the culture from different carbon sources: *L. sakei strains* with galactose and *E. coli* with glucose*.* Among the two ACE-βCs detected, higher amounts were generally found for AβC than for AβC-COOH.

**Fig. 3:**
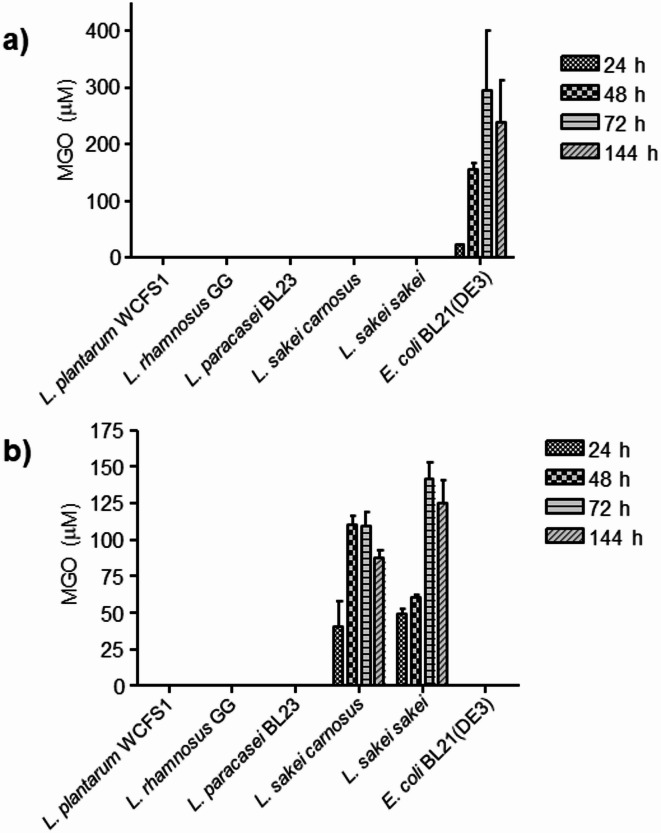
Presence of MGO (μM) in the supernatants of bacterial cultures grown in mMRS media containing glucose (2%) (**a**) or galactose (2%) (**b**). MGO was analyzed at 24, 48, 72 and 144 h of incubation.

The same microbial cultures were analyzed for the presence of MGO by HPLC–DAD (317 nm) and MS following its chemical derivatization to form 2-methylquinoxaline (Figures S3 and S4). The microbial cultures containing ACE-βCs like those from *E. coli* and *L. sakei* strains (Fig. 2) also contained MGO (Fig. 3). In contrast, MGO was very low or undetected in the rest of bacterial cultures including those from lactic acid bacteria other than *L. sakei*. Then, as seen in Figs. 2 and 3, the presence of ACE-βCs in the culture supernatants from *E. coli* and *L. sakei* strains (72 or 144 h) was accompanied by the presence of MGO in early stages of cultures (48–72 h) suggesting that the MGO generated by bacteria could subsequently led to ACE-βCs formation.

### MgsA in MGO-producing bacteria and formation of ACE-βCs

**Fig. 4:**
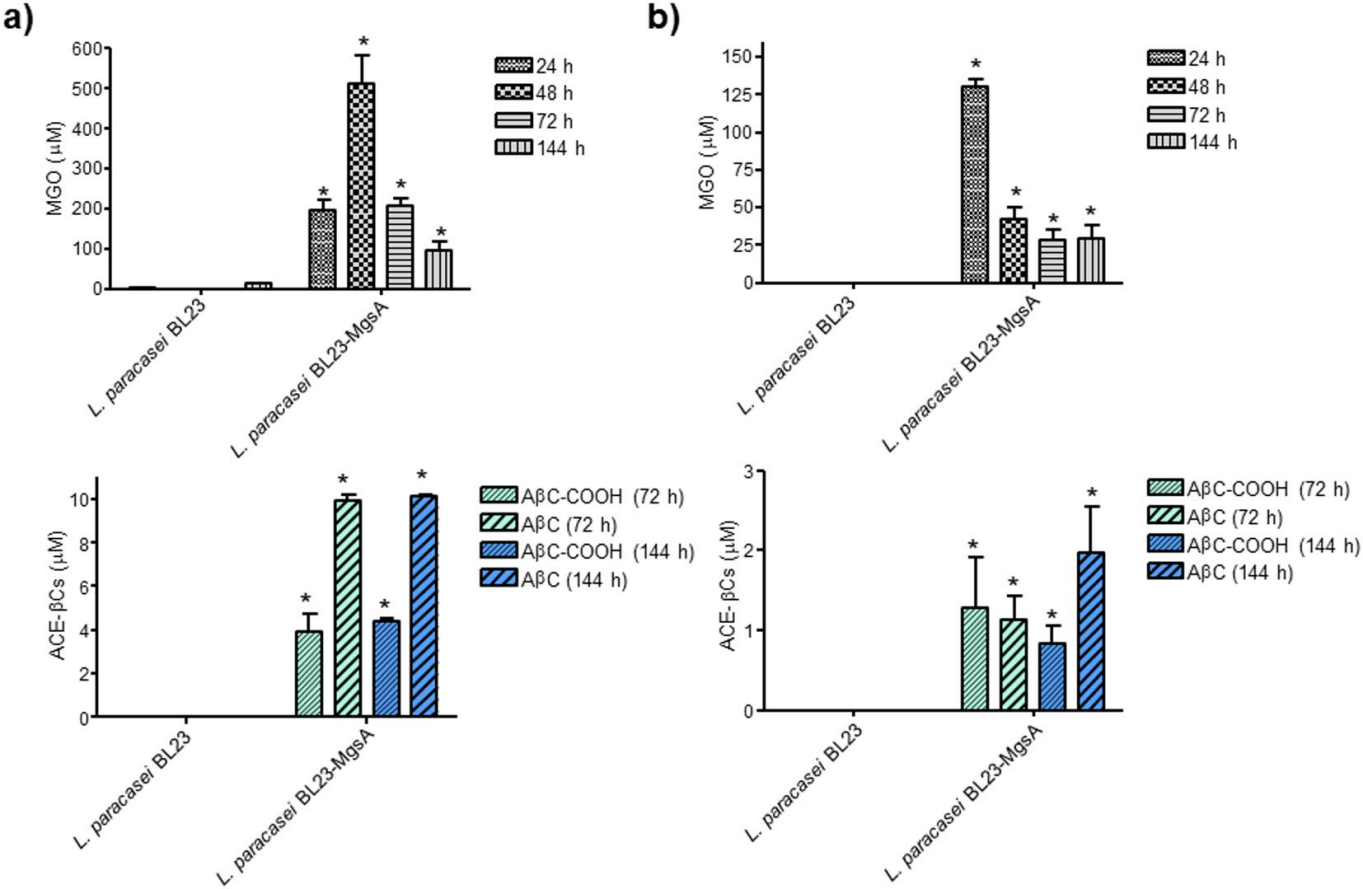
Presence of MGO (μM) and ACE-βCs (μM) in culture supernatants of *L. paracasei* BL23 and recombinant *L. paracasei* BL23-MgsA grown in mMRS media containing glucose (2%) (**a**) or galactose (2%) **(b**). MGO was analyzed at 24, 48, 72 and 144 h of incubation. ACE-βCs were analyzed at 72 and 144 h of incubation. (*) Results are significantly different (p < 0.05, Student´s t-test) from those corresponding to wild type. Data are mean ± SEM (n = 2). Control cultures of *L. paracasei* BL23 (pNZ:TuB) (plasmid without MgsA) did not produce MGO or ACE-βCs.

The enzyme methylglyoxal synthase (MgsA) that catalyzes the formation of MGO from dihydroxyacetone phosphate (DHAP) has been reported in *E. coli*^27,28^ and other bacteria but scarcely in lactic acid bacteria. A search in the complete genome databases of lactic acid bacteria revealed the presence of an enzyme annotated as “methylglyoxal synthase” (MgsA) in the genomes of *L. sakei* subsp. *carnosus* (accession WP_056948772.1) and *L. sakei* subsp. *sakei* (accession WP_010495548.1). Both proteins are 99.29% identical, and exhibited an identity degree of 47.41 with *E. coli* MgsA (Figure S5). However, MgsA is rare among lactic acid bacteria. Only 32-type strains out of more than 450 species of the *Lactobacillaceae* family contain the *mgsA* gene in their genome (Table 1). MgsA proteins from lactic acid bacteria are 35.20–49.67% identical to MgsA from *E. coli* (Table 1 and Figure S6). Interestingly, *L. plantarum*, *L. rhamnosus*, and *L. paracasei* species are not included among species possessing MgsA, and these strains did not produce appreciable amounts of MGO (Fig. 3). On the contrary, bacteria containing MgsA could synthetize MGO, and indeed, strains from *E. coli* and *L. sakei* species that contain MgsA (Figure S5) were able to produce MGO as seen in Fig. 3. These results suggested a link between MGO production by MgsA and ACE-βCs formation. In order to demonstrate this link, the *mgsA* gene from *L. sakei* subsp*. carnosus* DSM 15831^ T^ was introduced into *L. paracasei* BL23 strain, a non-MGO producer. When the recombinant strain *L. paracasei* BL23-MgsA (*L. paracasei* BL23 (pNZ:TuB.MgsA)) was grown in mMRS supplemented with glucose or galactose, it produced MGO and its culture supernatant contained ACE-βCs (Figs. 4 and S7). In contrast, the wild type *L. paracasei* BL23 strain lacking an *mgsA* gene copy did not produce MGO and their culture supernatants did not contain AβC and AβC-COOH. Therefore, these results confirmed that bacteria containing MgsA produced MGO that subsequently led to ACE-βCs formation, evidencing a direct link between MgsA, MGO and ACE-βCs.

**Table 1:** Type-strain from species of the *Lactobacillaceae* family possessing methylglyoxal synthase (MgsA)^1^.

Genera	Species/subspecies	Type strain	Accession	% Identity MgsA	
				*vs L.sakei*	*vs E.coli*
*Agrilactobacillus*	*A. composti*	DSM 18527^ T^	WP_035452778.1	75.71	48.89
	*A. fermenti*	CC-MHH1034^T^	WP_230914713.1	74.29	46.67
	*A. yilanensis*	54-2^ T^	WP_125715879.1	72.14	45.19
*Lacticaseibacillus*	*L. jixiensis*	N163-3-2^ T^	WP_390410663.1	70.71	41.48
	*L. kribbianus*	YH-lac21^T^	WP_225047161.1	67.14	42.22
	*L. mingshuiensis*	117-1^ T^	WP_203626981.1	67.14	45.19
	*L. nasuensis*	DSM 26653^ T^	WP_056951331.1	66.43	44.44
	*L. parakribbianus*	YH-lacS6^T^	WP_262314705.1	65.00	40.71
	*L. saniviri*	DSM 24301^ T^	WP_056992424.1	65.44	41.48
	*L. yichunensis*	33-1^ T^	WP_125696967.1	66.43	44.44
*Lactiplantibacillus*	*L. herbarum*	TCF032-E4^T^	WP_047999447.1	43.20	35.20
*Latilactobacillus*	*L. curvatus*	DSM 20019^ T^	WP_004269993.1	90.00	46.67
	*L. graminis*	DSM 20719^ T^	WP_057908374.1	89.29	46.67
	*L. fragifolli*	AMBP162^T^	WP_154241818.1	90.71	46.67
	*L. fuchuensis*	DSM 14340^ T^	WP_025083183.1	90.00	49.63
	*L. sakei* subsp*. carnosus*	DSM 15831^ T^	WP_056948772.1	100.00	47.41
	*L. sakei* subsp*. sakei*	DSM 20017^ T^	WP_011374855.1	99.29	47.41
*Ligilactobacillus*	*L. acidipiscis*	DSM 15353^ T^	WP_010495548.1	67.86	46.67
	*L. pobuzihii*	DSM 28122^ T^	WP_017867647.1	66.43	47.41
	*L. salitolerans*	DSM 103433^ T^	WP_124974693.1	67.86	45.19
*Liquorilactobacillus*	*L. sicerae*	CUPV261^T^	WP_281165630.1	66.91	44.44
	*L. vini*	DSM 20605^ T^	WP_010579891.1	68.38	44.44
*Loigolactobacillus*	*L. bifermentans*	DSM 20003^ T^	WP_057904775.1	63.57	44.44
	*L. binensis*	735-2 T	WP_137638180.1	67.14	39.26
	*L. coryniformis* subsp*. coryniformis*	DSM 20001^ T^	WP_003677980.1	66.43	41.96
	*L. coryniformis* subsp*. torquens*	DSM 20004^ T^	WP_010013276.1	67.14	42.96
	*L. jiayinensis*	257-1^ T^	WP_125551858.1	70.71	42.22
	*L. rennini*	DSM 20253^ T^	WP_057873700.1	66.43	43.70
	*L. zhaouyuanensis*	187-3^ T^	WP_125549207.1	65.71	39.26
*Schleiferilactobacillus*	*S. harbinensis*	DSM 16991^ T^	WP_027828140.1	73.91	47.41
	*S. perolens*	DSM 12744^ T^	WP_057822178.1	73.91	44.44
	*S. shenzhenensis*	LY-73^ T^	WP_022529735.1	71.01	47.41

^1^The List of Prokaryotic names with Standing in Nomenclature (LPNS) (https://lpsn.dsmz.de/) was used to search into the *Lactobacillaceae* family the genera with a validly published name under the ICNP (International Code of Nomenclature of Prokaryotes). Then, the LPNS was also used to search the species on each genera with a validly published name under the ICNP. Finally, the Taxonomy Browser tool of the NCBI page (https://www.ncbi.nlm.nih.gov/datasets/taxonomy/tree/) was used to search for the presence of a protein annotated as MgsA (methylglyoxal synthase) on the type strain of each one of the 450 species with a validly published name identified in the *Lactobacillaceae* family.

**Fig. 5:**
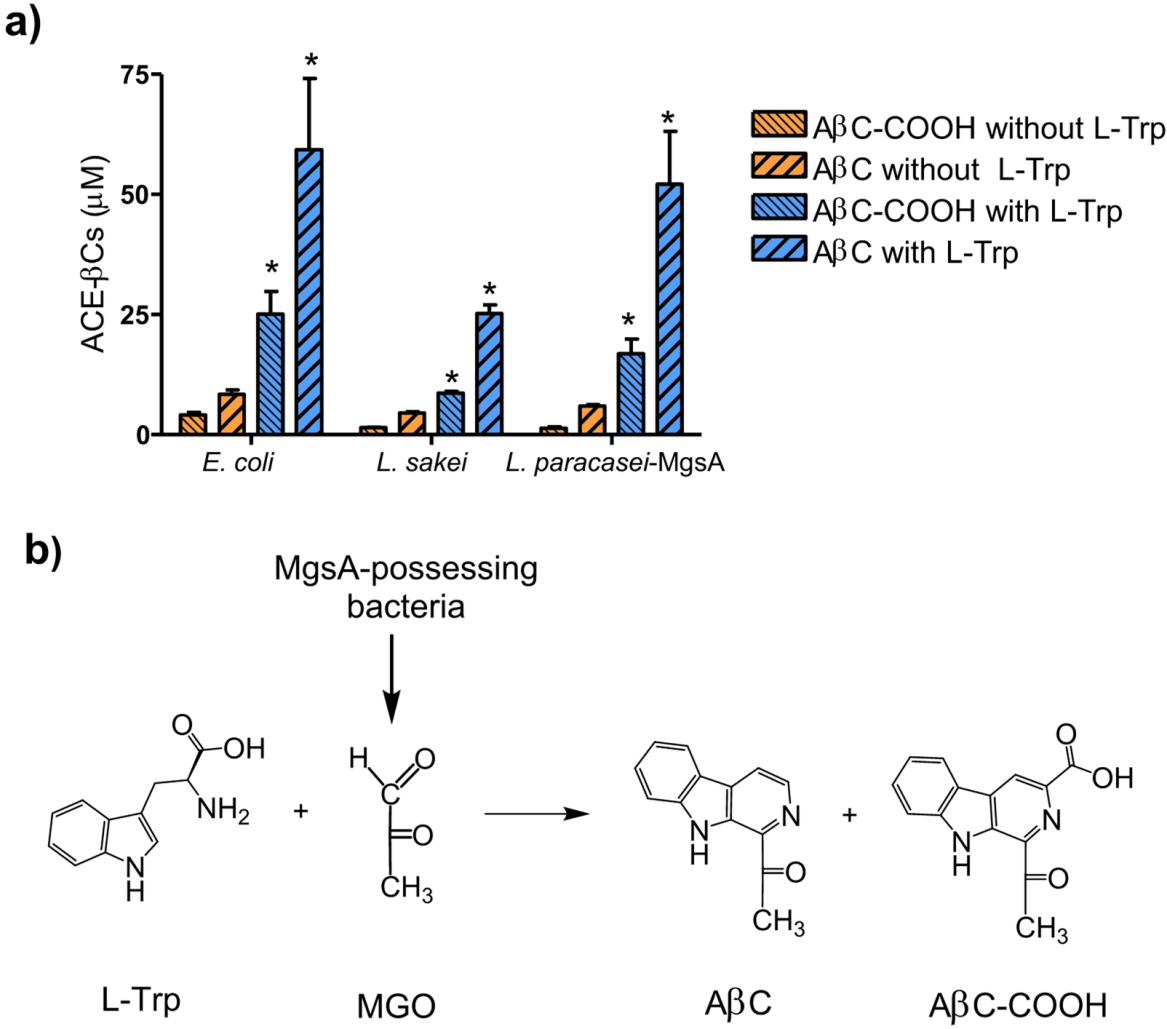
Presence of ACE-βCs in supernatants of bacterial cultures grown for 144 h in mMRS media or in mMRS supplemented with L-Trp (0.6 mg/mL). *E.coli* BL21(DE3) and *L. paracasei* BL23-MgsA were grown in media containing glucose (2%) and *L. sakei* subsp. *carnosus* DSM 15831^ T^ grown in media containing galactose (2%). (*) Results are significantly different (p < 0.05, Student´s t-test) from without L-Trp. Results are mean ± SEM from triplicates (**a**). Schematic representation of the spontaneous reaction of L-Trp with MGO produced by MgsA-possessing bacteria to form ACE-βCs (**b**).

Therefore, MGO produced by bacteria could led to ACE-βCs formation. Since MGO reacts with L-Trp to form ACE-βCs^21,22^ L-Trp was added to MGO-producing strains. When L-Trp was added to mMRS media containing glucose (for *E. coli* and *L. paracasei* BL23-MgsA) or galactose (for *L. sakei* subsp. *carnosus*) and incubated, the formation of AβC-COOH and AβC, increased substantially (Fig. 5a). In addition, it was previously demonstrated that ACE-βCs formation increased when external MGO was added to MRS media^21.^ These results confirmed that MGO produced by bacteria reacts spontaneously with L-Trp present in the media to form AβC and AβC-COOH (Fig. 5b). The mechanism of this reaction has been recently studied in foods where MGO mainly arise from carbohydrate degradation^22^.

### Factors influencing MGO production in MgsA-possessing bacteria.

**Fig. 6:**
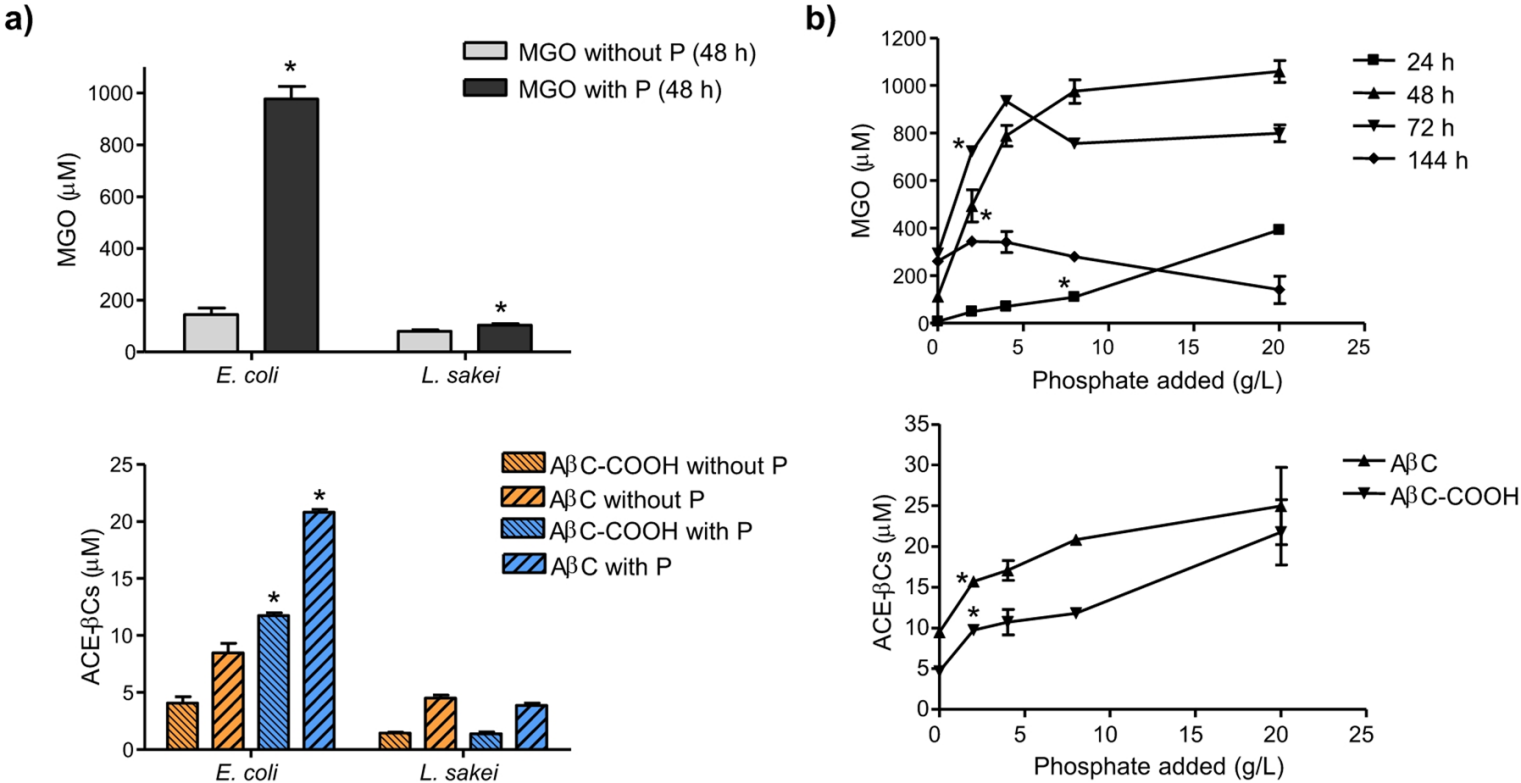
Presence of MGO and ACE-βCs in supernatants of bacterial cultures grown in mMRS media or in mMRS supplemented with dipotassium phosphate (8 mg/mL). *E.coli* BL21(DE3) was grown in media containing glucose (2%) and *L. sakei* subsp. *carnosus* DSM 15831^ T^ grown in media containing galactose (2%) (**a**). (*) Results are significantly different (p < 0.05, Student´s t-test) from absence of added phosphate. Presence of MGO and ACE-βCs in *E. coli* BL21(DE3) culture supernatants incubated for 144 h in mMRS media containing glucose (2%) and increasing concentrations of dipotassium phosphate (0–20 g/L) (**b**). The one-way ANOVA indicated significant effect of phosphate on MGO (F(4,6) = 121.3, P < 0.0001; (F (4,7) = 36.11, P < 0.0001, and (F(4,7) = 119.7, P < 0.0001) for 24, 48 and 72 h, respectively) and ACE-βCs: AβC (F(4,10) = 19.37, P < 0.0001) and AβC-COOH (F(4,10) = 25.08, P < 0.0001). (*) Results of MGO (P < 0.01) and ACE-βCs (P < 0.05) are significantly different (Dunnett´s post test) from the absence of added phosphate for this and higher concentrations of phosphate added. MGO determinations were taken at 24, 48, 72 h and 144 h of incubation and ACE-βCs were determined at 144 h. Results are mean ± SEM from duplicate or triplicate experiments.

The factors influencing the metabolic production of MGO by MgsA-possessing bacteria and the formation of ACE-βCs were subsequently studied under different growth conditions regarding the carbohydrate used, phosphate and aeration conditions. As mentioned above, bacteria without MgsA did not produce MGO with either glucose or galactose (and neither led to ACE-βCs formation). Among those bacteria possessing MgsA, Fig. 3 showed that *E. coli* only produced MGO (which led to ACE-βCs formation) from glucose but not from galactose, whereas the two strains of *L. sakei* mainly produced MGO from galactose. This suggested the existence of different metabolic pathways and regulation of carbohydrate metabolism in MgsA-containing bacteria. The formation of MGO by MgsA-possessing bacteria was subsequently studied in mMRS supplemented with phosphate. Interestingly, MGO production by *E. coli* increased substantially when cultures contained phosphate added, and ACE-βCs formation followed the same trend (Fig. 6a). Thus, the MGO production was about sevenfold higher in the presence of phosphate added (8 g/L). The presence of phosphate also increased MGO production in *L. sakei* but to a lesser extent whereas ACE-βCs formation was not significantly different. Since the addition of phosphate clearly influenced the formation of MGO in *E. coli* (Fig. 6a), the synthesis of MGO was studied as a function of phosphate concentration under a fixed concentration of glucose in the media. Noticeably, the production of MGO in the incubation period from 24 to 72 h increased substantially with the addition of phosphate (Fig. 6b), and this increase was not directly correlated with growth or affected by pH (Figure S8). The MGO content in the culture supernatants decreased in prolonged incubation times (144 h) likely due to chemical reaction and/or degradation. The presence of ACE-βCs in the culture supernatants followed the same trend and also increased with the addition of phosphate (Fig. 6b).

**Fig. 7:**
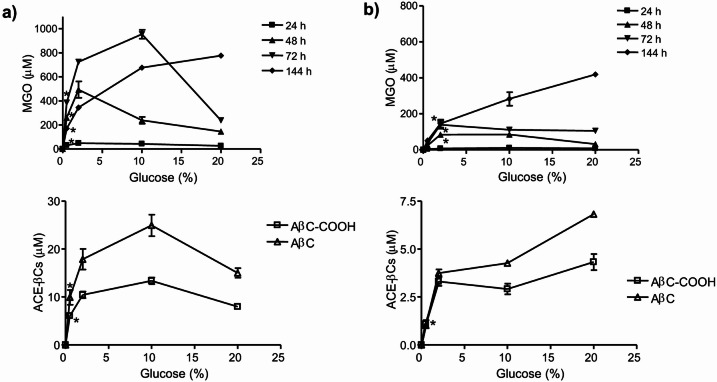
Presence of MGO and ACE-βCs in *E. coli* BL21(DE3) culture supernatants grown in mMRS media containing increasing concentrations of glucose (0–20%) with dipotassium phosphate added (2 g/L) (**a**), and in the same media but without phosphate added (**b**). MGO determinations were taken at 24, 48, 72 h and 144 h of incubation. ACE-βCs were determined at 144 h of incubation. (**a**) The one-way ANOVA indicated significant effect of glucose on MGO (F (4,10) = 30.05, P < 0.0001, F(4,10) = 390.8, P < 0.0001) and F(4,10) = 670.5, P < 0.0001 for 48, 72 and 144 h, respectively) and on ACE-βCs: AβC (F(4,10) = 33.53, P < 0.0001) and AβC-COOH (F(4,10) = 93.12, P < 0.0001). (*) Results of MGO and ACE-βCs are significantly different (p < 0.01) (Dunnett´s post test) from absence of glucose for this (0.5%) and higher concentrations of glucose. (**b**) The one-way ANOVA indicated significant effect of glucose on MGO (F(4,10) = 57.43, P < 0.0001; F(4,10) = 29.59, P < 0.0001; (F(4,10) = 72.70, P < 0.0001 for 48, 72 and 44 h, respectively), and on ACE-βCs: AβC (F(4,9) = 502.7, P < 0.0001) and AβC-COOH (F(4,9) = 52.54, P < 0.0001). (*) Results of MGO and ACE-βCs are significantly different (P < 0.01) from absence of glucose for this and higher concentrations of glucose. Results are mean ± SEM from triplicates.

**Fig. 8:**
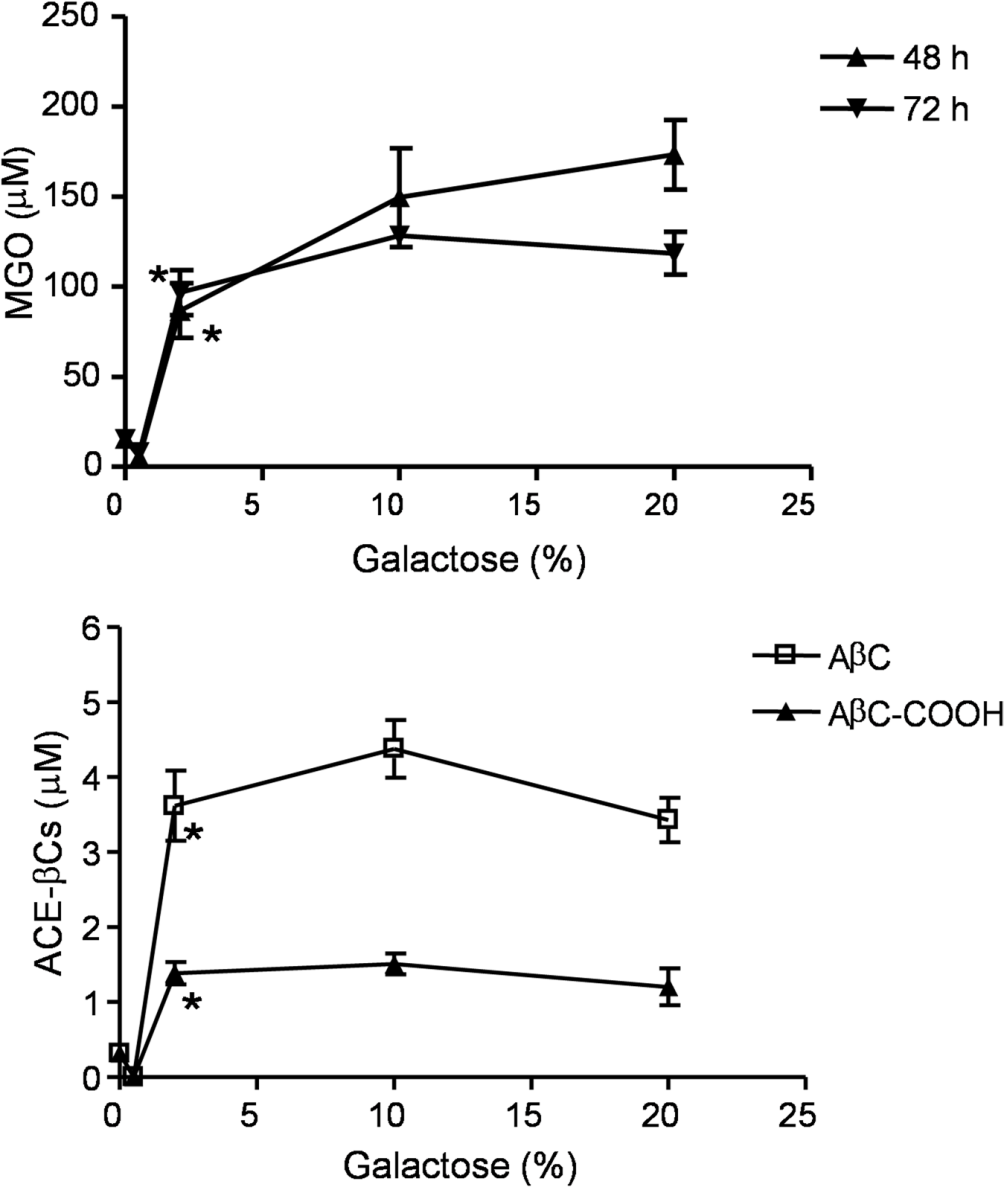
Presence of MGO and ACE-βCs in *L. sakei* subsp. *carnosus* DSM 15831^ T^ culture supernatants grown in mMRS media containing increasing concentrations of galactose (0–20%). MGO determinations were taken at 48 and 72 h of incubation. ACE-βCs were determined at 144 h of incubation. Results are significant (one-way ANOVA) for MGO (F(4,15) = 21.27, P < 0.0001) (48 h), (F(4,12) = 27.83, P < 0.0001) (72 h), AβC (F(4,10) = 17.26, P < 0.0001) and AβC-COOH (F(4,10) = 11.96, P < 0.0001). (*) Results are significantly different (p < 0.01, Dunnett´s test) from absence of galactose at 2% and higher concentrations of galactose. Results are mean ± SEM from triplicates.

The synthesis of MGO by MgsA-possessing bacteria was subsequently studied under increasing concentrations of sugar. MGO formation in *E. coli* occurred after 24 h of growth and highly increased with the addition of glucose (Fig. 7a). ACE-βCs formation also increased with the addition of glucose as occurred with MGO levels. Noticeably, in the absence of glucose, *E. coli* did not produce MGO (with no ACE-βCs detected in the supernatants) despite showing a similar growth (Figure S9). As expected from results described above, higher amounts of MGO and ACE-βCs were found in the bacterial cultures when phosphate was added (Fig. 7a) as compared with those without phosphate (Fig. 7b). Regarding *L. sakei*, the MGO production (and the resultant ACE-βCs) significantly increased with the increase of galactose in the medium, and very low levels were found in low concentrations of galactose (Fig. 8). These results suggest that the carbohydrate and its concentration are key factors for the synthesis of MGO by MgsA-containing bacteria, and the subsequent formation of ACE-βCs.

**Fig. 9:**
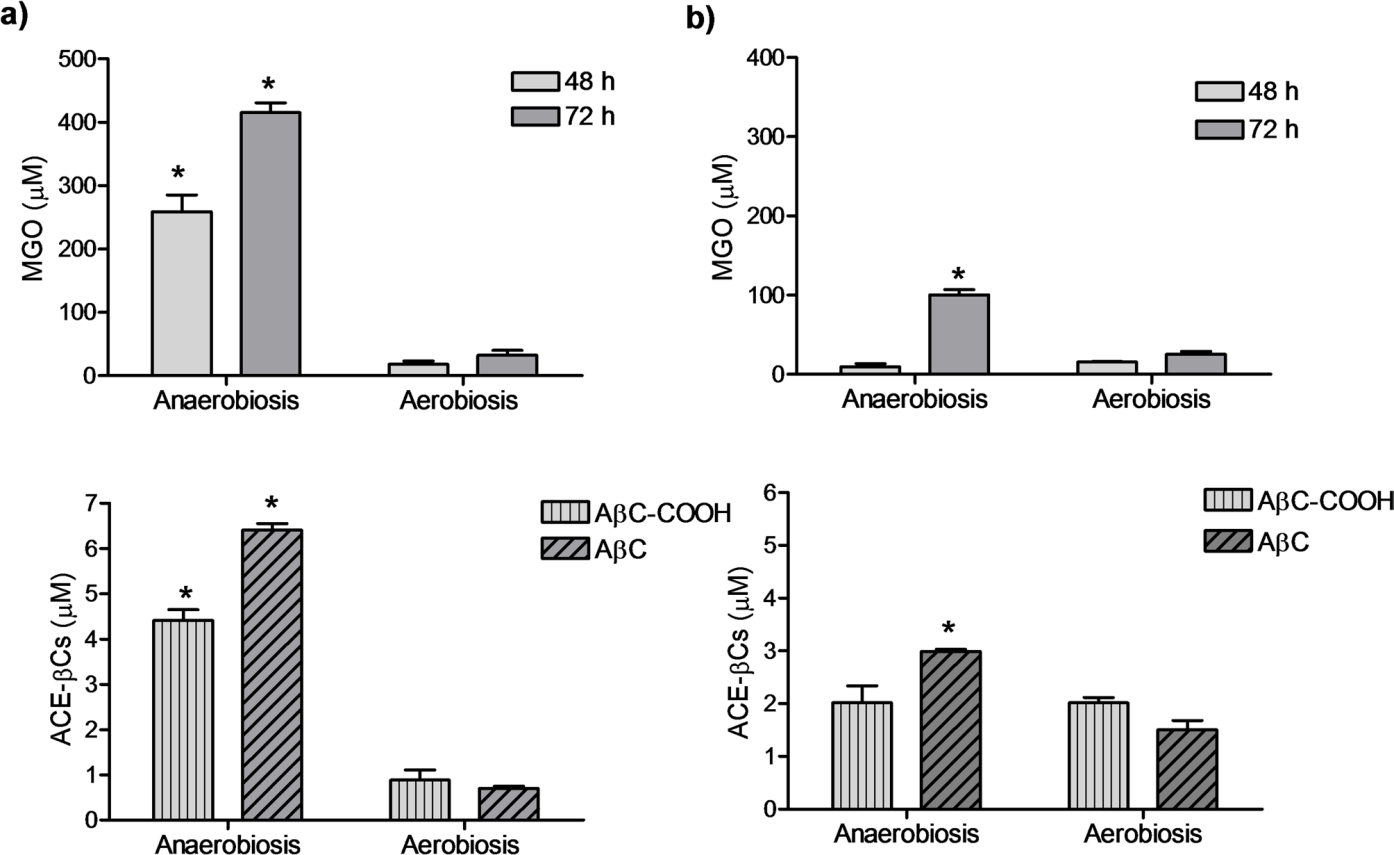
Presence of MGO and ACE-βCs in *E. coli* BL21(DE3) culture supernatants grown in mMRS media containing glucose (2%) and dipotassium phosphate added (2 g/L) (**a**) or in mMRS media containing glucose (2%) without phosphate added (**b**) in anaerobic as well as in aerobic (high oxygen) conditions. MGO determinations were taken at 48 and 72 h of incubation. ACE-βCs were determined at 144 h of incubation. (*) Differences are significant (p < 0.05, Student´s t-test) between anaerobic and aerobic growth. Results are mean ± SEM from duplicate experiments.

Finally, *E. coli* was grown in mMRS media containing glucose under anaerobiosis or instead under high aeration conditions (Erlenmeyer-flaks under agitation) and the presence of MGO and ACE-βCs was determined (Fig. 9). The absence of oxygen did not avoid the production of MGO whereas high aeration conditions highly decreased the formation MGO. The synthesis of MGO was significantly higher under absence of oxygen (anaerobiosis) than under high oxygen conditions (aerobiosis) (Fig. 9a). Although considerable bacterial growth occurred under high oxygen conditions (aeration) (Figure S10), very low MGO was produced under these conditions (Fig. 9a)**.** In contrast, relatively high levels of MGO were produced under anaerobiosis despite a relative low bacterial growth (Figure S10). As observed in previous assays, the formation of ACE-βCs correlated with the MGO content (Fig. 9) and the concentration of these compounds was generally higher in presence of phosphate added than without phosphate (Fig. 9ab).

## Discussion

Metabolites produced by bacteria are relevant in the human health and food science. Specifically, lactic acid bacteria play a beneficial role in both areas. The role of these bacteria in food fermentation has been known for a long time. More recently, lactobacilli present in the microbiota have been increasingly involved in important biochemical transformations^29,30^. A previous research has described that several lactobacilli species produced the bioactive alkaloid 1-acetyl-β-carboline (AβC) but these results have not been confirmed later^21,23^. Nevertheless, it was hypothesized that AβC might arise from MGO purportedly present in the culture media^21^. Thus, bacteria might produce MGO that could subsequently led to ACE-βCs. This work confirms now this hypothesis by assessing the presence of MGO and ACE-βCs in the culture supernatants of several lactobacilli and *E. coli*. There was a correlation between the presence of MGO and ACE-βCs. The results show the enzymatic production of MGO by some bacterial species and its subsequent non-enzymatic reaction (spontaneous) with L-Trp in the media to give ACE-βCs. As a result, two βCs were detected and characterized as 1-acetyl-β-carboline (AβC) and 1-acetyl-β-carboline-3-carboxylic acid (AβC-COOH). Formation of AβC was higher than AβC-COOH likely due to the acidic pH of the media (pH around 5) as a consequence of bacterial growth^21,22^.

The presence of MGO in the bacterial supernatants mainly depended on the bacteria involved. Lactic acid bacteria from the *Lactobacillaceae* family such as *L. plantarum, L. rhamnosus and L. paracasei* did not produce MGO and consequently did not afford ACE-βCs (AβC and AβC-COOH). In contrast, MGO and ACE-βCs were detected in culture supernatants from *L. sakei * and *E. coli.* Microorganisms containing the enzyme methylglyoxal synthase (MgsA)^31^ are able to produce MGO enzymatically. A search in the NCBI Taxonomy Browser genome databases indicate that bacteria from *E. coli* and *L. sakei* species contain the enzyme MgsA and shows similarity among them (46.38% identity) (Figure S6 and Table 1). Only a reduced number of species from the family *Lactobacillaceae* contain MgsA (32 out 450 type strains) (Table 1 and Figure S6); therefore, with a few exceptions, most lactic acid bacteria species from this family do not contain MgsA, and therefore cannot synthetize MGO. Consequently, the production of MGO by lactobacilli and the resultant ACE-βCs formation is unlikely^21^. In contrast, those microorganisms from *Lactobacillaceae* family possessing the MgsA enzyme (Table 1) could produce MGO enzymatically and subsequently afford ACE-βCs by a non-enzymatic reaction with L-Trp. To prove this assumption, the MgsA-encoding gene from *L. sakei* subsp. *carnosus* DSM 15831^ T^ (Figure S5), which produced MGO was cloned in *L. paracasei* BL23, a species unable to produce MGO. The recombinant strain obtained *L. paracasei* BL23*-*MgsA, was able to produce MGO, and subsequently afforded ACE-βCs. In contrast, the native *L. paracasei* BL23 wild type strain (without an *mgsA* gene copy) did not produce MGO (nor afforded ACE-βCs). These results proved the involvement of methylglyoxal synthase (MgsA) in the production of MGO by MgsA-containing bacteria like *L. sakei* and *E. coli* and the subsequent formation of ACE-βCs by a non-enzymatic reaction. This finding is also in agreement with a previous report of a *L. casei* strain engineered with MgsA from *Thermoanaerobacterium thermosaccharolyticum* HG-8, which was used to produce Parmesan cheese and increased MGO production and heterocyclic amines content^32^.

**Fig. 10:**
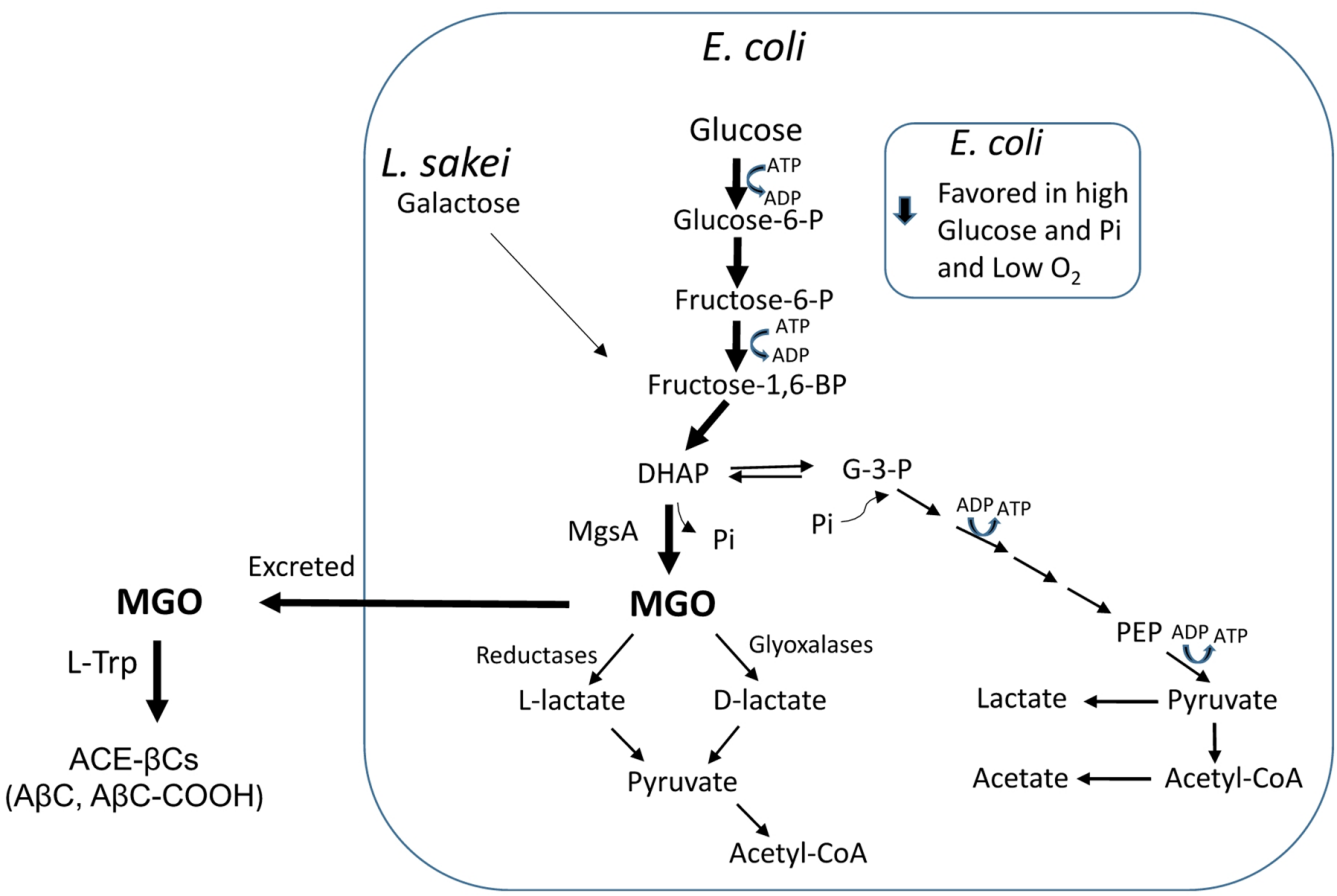
Scheme of the metabolism of glucose by *E. coli* or galactose by *L. sakei* in the glycolysis and biosynthesis of methylglyoxal (MGO) from dihydroxyacetone phosphate (DHAP) by methylglyoxal synthase (MgsA) in the so-called MGO bypass. The MGO can be metabolized by glyoxalases or ketoreductases or be excreted into the medium where it reacts spontaneously with L-Trp and affords ACE-βCs alkaloids. Production of MGO is favoured in the presence of high concentrations of carbohydrates and phosphate, and limited oxygen in *E. coli* and higher concentration of galactose in *L. sakei.* Glyceraldehyde-3-phosphate (G-3-P), phosphoenolpyruvate (PEP), and phosphate (Pi).

Results of this study have shown that media composition and growth conditions affected the production of MGO. MGO was assayed when carbohydrates or phosphate were added to the culture media. When *E. coli* was grown in mMRS medium, no MGO production was detected; however MGO was produced when glucose (but not galactose) was added. Then, the presence of glucose was required to produce MGO by *E. coli*. Since MGO arises from dihydroxyacetone phosphate (DHAP) during glycolysis by the action of MgsA (Fig. 10), it is expected that enhanced bacterial growth using glucose can generate more phosphorylated precursors, and particularly DHAP, which will be converted into MGO by MgsA. Interestingly, higher production of MGO occurred with increased levels of glucose. Moreover, MGO production increased under anaerobic vs aerobic conditions. In this regard, the growth in the absence of oxygen (anaerobic conditions) is expected to increase the rate of glucose used in glycolysis, which could turn in more synthesis of MGO. Thus, under oxygen limitations (anaerobic or oxygen-limited conditions), *E. coli* metabolism favors the use of glucose in glycolysis leading to more phosphorylated intermediates and MGO. Contrarily, under high oxygen conditions (aeration), *E. coli* largely decreased production of MGO. This occurred even in the presence of glucose and phosphate added, whereas the pH in the cultures decreased (pH around 5), suggesting the formation of acetate^33,34^. It has been described that in aeration and presence of glucose, *E. coli* changes its metabolism to respiro-fermentative acetate pathway (overflow metabolism) to get more energy from glucose^35^. Therefore, under these conditions the production of MGO from glucose will be limited (Fig. 10).

Contrary to *E. coli*, *L. sakei* produced MGO in mMRS media when galactose was added but not with glucose. The production of MGO by *L. sakei* increased as the concentrations of galactose increased. Lactic acid bacteria metabolize galactose by Leloir and/or tagatose routes^36,37^ and these pathways can led to DHAP and then MGO production if the bacteria contain MgsA (Fig. 10). It is unknown whether *L. sakei* uses the tagatose pathway or its involvement in the MGO production. MgsA is induced in *L. sakei* when grown in D-ribose^38^ vs glucose, and it could be a response to the adaptation to particular growth conditions like glucose starvation^39^.

The role of MGO in bacteria is a controversial issue owing to its toxic nature and because it can modulate cell survival and toxicity^11,40.^ MgsA catalyzes the conversion of DHAP into MGO and inorganic phosphate (Fig. 10). This enzyme plays a significant role in bacterial metabolism as a distinct pathway in glycolysis. Thus, the MgsA provides an alternative pathway to triosephosphate isomerase (TPI) for metabolizing DHAP in bacteria, called methylglyoxal bypass (Fig. 10) ^10^. MgsA can be involved in the regulation of glycolysis and the adaptation to stress conditions. Results in this work and elsewhere confirm that its activity is influenced by factors such as the availability of a specific carbon source and the metabolic flux through glycolysis^41^. Interestingly, our results also evidence, for the first time, that the growth of *E. coli* under increasing concentrations of phosphate highly increased the metabolic production of MGO. These results are noticeable as suggest that bacteria under high phosphate levels may increase the phosphorylated intermediates (glucose-6-P and fructose-6-P, fructose-1,6-BP and DHAP) in glycolysis triggering the production of MGO (Fig. 10). In a previous study, *E. coli* grown in presence of glucose-6-P or fructose-6-P increased MGO production and resulted in inhibition of bacterial growth^42^. On the other hand, it has been reported that the MgsA activity is inhibited by phosphate suggesting that bacteria may use this mechanism to regulate phosphate intracellular levels^27^. However, results in this work show that the presence of phosphate in the culture media did not inhibit but instead increased MGO production in *E. coli.*

Therefore, carbohydrates, phosphate and oxygen (aeration) determined the production of MGO in *E. coli*. Under oxygen limitation, *E. coli* produced MGO from glucose and higher concentrations of glucose and/or phosphate increased MGO production (Fig. 10). However, under high oxygen (aerobic conditions) or absence of glucose, *E. coli* greatly reduced the synthesis of MGO. The metabolic production of MGO has been considered a paradox due to its toxic nature and because it might allow bacteria to adapt to certain environmental conditions and nutrient imbalance^10,13^. The MGO production might relieve bacteria from the stress caused by phosphorylated intermediates in glycolysis while offering survival advantage^11,12,31,42^. In fact, the phosphorylated intermediates are toxic for *E. coli* and caused depletion of key glycolytic intermediates downstream^11,42,43^. Therefore, taken together, our result suggest that under abundant glucose, phosphate and limitation of oxygen, the production of MGO may be an strategy for *E. coli* to control the rate of carbon (glucose) flux in glycolysis, and reduce the accumulation of toxic phosphorylated intermediates (survival strategy) (Fig. 10). This is in line with the fact that MGO was mainly excreted after 24 h of bacterial growth. Although MGO is toxic and accumulates when the production overcomes the capacity of detoxification; bacteria can remove MGO by the action of enzymes such as glyoxalases (glutathione dependent glyoxalase I-II system) and ketoreductases^3,7,8,44^. In our conditions, up to 2.5 mM added MGO was needed for inhibition of *E. coli* BL21 growth (minimal inhibitory concentration) whereas the MGO produced was excreted into the media during bacterial growth and decreased during prolonged incubation (144 h) (Fig. 6), what may be also an strategy against its toxicity (Fig. 10).

In the human body, MGO is released as a non-enzymatic byproduct of triosephosphates during glycolysis^9^. An estimated 0.1–0.4% of the glycolytic flux results in MGO production^45^. MGO reacts with proteins, lipids and nucleic acids altering their function and is a precursor of advanced glycation end products (AGEs) involved in the pathogenesis of age-related diseases^4,6,9^. Elevated levels of MGO and AGEs have been observed in individuals with diabetes, vascular complications, inflammation and chronic diseases^1–5^. MGO is also present in foods arising during food processing^46–49^. MGO could be also produced by human gut microbiota, and studying its impact on the host health is an emerging topic of research^13,14,50^. The results reported here have shown that bacteria possessing MgsA such as *L. sakei* from *Lactobacillaceae* family (Table 1 and Figure S6) and *E. coli* produce MGO. As shown here, MGO derived from these bacteria reacts with L-Trp to form ACE-βCs alkaloids. Therefore, the presence of ACE-βCs could be a reporter of bacterial MGO*.* Lactic acid bacteria possessing MgsA, such as *L. sakei*, when present in foods fermentation (*e.g.* meats)^39,51^ could produce MGO, and then result in ACE-βCs formation. In addition, bacteria in the human gut microbiota such as *E. coli* and others containing MgsA like those from *Lactobacillaceae* family (Table 1) can generate MGO resulting in ACE-βCs formation. Indeed, considering the result of this study, *E. coli* is expected to produce MGO from glucose in the human gut (limited oxygen available). The MGO produced by *E. coli* or other MgsA-possessing bacteria in the gut may affect the host. In a previous study, the metabolism of glucose by *E. coli* affected the healthspan and lifespan of the host *Caenorhabditis elegans*^52^. Contrarily, bacteria that do not possess MgsA including most lactic acid bacteria are not expected to produce MGO and the presence of ACE-βCs as a result, will be negligible. In this regard, previous studies reporting that lactic acid bacteria in microbiota which do not have MgsA produced ACE-βCs should be revised in line with this work^21–24,53^. Trace levels of ACE-βCs may have formed by a spontaneous reaction of L-Trp with MGO derived from carbohydrate degradation^21,22^.

βC alkaloids are naturally-occurring bioactive alkaloids that interact with the CNS and inhibit key enzymes such as MAO and kinases showing antidepressant, neuroprotective, anticancer, antimicrobial and antioxidant activities^15–17,19,54^. These compounds can be also bioactivated to neurotoxic *N*-methyl-β-carbolinium cations^20,55^. Numerous βCs have been identified in foods including carbohydrate-derived βCs and βCs arising from α-dicarbonyl compounds such as ACE-βCs, perlolyrine and flazin^22,56–60^. So far, little is known on the specific biological activity of ACE-βCs. AβC inhibits *Candida albicans* filamentation due to inhibition of DYRK1 kinase suggesting that βCs could be good scaffolds against *Candida* diseases^23^. In a recent study, AβC has shown anti-skin cancer potential^53^. Analogs of ACE-βCs from deep sea organisms such as the actynomycete *Marinactinospora thermotolerans* have been evaluated as promising anticancer agents^61,62^ and identified as potent anti-inflammatory substances^24^. AβC-COOH has been recently reported as a plant growth regulator and a photosynthesis enhancer^63^. ACE-βCs could be also considered a new type of advanced glycation end-products (AGE) derived from MGO, and their formation in vivo (*e.g.* from microbiota containing MgsA) may contribute to scavenging toxic MGO^22^. In this regard, the formation of ACE-βCs might play a role in the reported effects of MGO related to tryptophan depletion^64^.

The results obtained here are focused on the bacterial formation of MGO (and the subsequent formation of ACE-βCs) and come from experiments in vitro. Although they support conceptually and experimentally the production of MGO (and ACE-βCs) by bacteria containing MgsA under gut conditions, further in vivo studies and/or with simulated gut models under controlled conditions will be needed to determine the extent and importance of this generation of MGO. These studies might address the possible implications of the MGO production and metabolism in the toxicity and bioactivity in the host. In addition, further studies may also address the implications of the biosynthesis of MGO in the toxicity or survival of MgsA-containing bacteria by studying intracellular and extracellular MGO levels. Finally, specific studies on the biological activity of ACE-βC alkaloids are also needed.

## Conclusions

This work reports the presence of MGO and bioactive ACE-βCs alkaloids in culture supernatants from *E. coli* and lactic acid bacteria. Two βCs were identified and characterized as AβC and AβC-COOH. The cultures containing ACE-βCs also contained methylglyoxal (MGO). It is shown the bacterial formation of MGO and its subsequent spontaneous reaction with L-Trp in the media to give ACE-βCs. The production of MGO occurred in microorganisms possessing the enzyme MgsA such as E*. coli* and *L. sakei.* In contrast, most lactic acid bacteria such as *L. plantarum*, *L. rhamnosus* or *L. paracasei* did not produce MGO, and therefore their cultures did not contain ACE-βCs. The involvement of MgsA was confirmed by introducing an MgsA-encoding gene into *L. paracasei* and the recombinant bacteria obtained produced MGO. The factors involved were evaluated and several conclusions obtained. First, the bacterial production of MGO depended on the carbohydrate present and its concentration. *E. coli* produced MGO with glucose whereas *L. sakei* produced MGO from galactose. Second, the MGO production increased as the concentration of carbohydrate (glucose or galactose) increased*.* Third, the MGO production by *E. coli* increased as the concentration of phosphate increased. This fact could be due to a higher accumulation of phosphorylated intermediates resulting in higher formation of MGO. Finally, the growth of *E. coli* under low oxygen conditions resulted in higher formation of MGO (and ACE-βCs). This fact suggest that *E. coli* may produce MGO from glucose in the human gut (low oxygen conditions). Taken together, these results indicate that factors increasing glycolysis flux such as carbohydrate concentration, phosphate and anaerobiosis determined the bacterial production of MGO. Bacteria possessing MgsA from foods or gut microbiota can produce MGO and this metabolite leads to formation of ACE-βCs. The production of MGO in foods and gut microbiota is a matter of interest since MGO is a reactive and toxic metabolite whereas ACE-βCs are bioactive substances being investigated in different targets.

## Supplementary Information

Below is the link to the electronic supplementary material.

Supplementary Material 1

## Data Availability

All data generated or analyzed during this study are included in this published article (and its Supplementary Information file).
