# Supplementary material for: Bacteria from foods and gut microbiota produce methylglyoxal and this metabolite leads to the formation of bioactive 1-acetyl-β-carboline alkaloids
Source: Sci Rep. 2026 Jan 9;16:4905. doi: 10.1038/s41598-026-35162-9 (PMC12873402; doi:10.1038/s41598-026-35162-9)
Supplement: Supplementary file 1 — Supplementary Material 1 [file 41598_2026_35162_MOESM1_ESM.docx]

**Supplementary information**

**Bacteria from foods and gut microbiota produce methylglyoxal and this metabolite leads to the formation of bioactive 1-acetyl-β-carboline alkaloids**

Tomás Herraiz^1^*, Ana Sánchez-Arroyo^1^, Blanca de las Rivas^1^, José María Landete^2^, Rosario Muñoz^1^.

^1^Instituto de Ciencia y Tecnología de Alimentos y Nutrición, ICTAN-CSIC. C/ José Antonio Nováis 6, Madrid, Spain

^2^Instituto Nacional de Investigación y Tecnología Agraria y Alimentaria, INIA-CSIC, Carretera de La Coruña km 7.5 Madrid, Spain

Table S1. Bacterial strains used in this work.

| Strain | Relevant phenotype | Source/  Reference |
| --- | --- | --- |
| *Escherichia coli* BL21(DE3) | MgsA+ | Invitrogen |
| *Lactilactobacillus sakei* subsp. *carnosus* DSM 15831^T^ | MgsA+ | DSMZ |
| *Lactilactobacillus sakei* subsp. *sakei* DSM 20017^T^ | MgsA+ | DSMZ |
| *Lacticaseibacillus rhamnosus* GG |  | DSMZ |
| *Lactiplantibacillus plantarum* WCFS1 |  | Kleerebezem et al., 2003 |
| *Lactococcus cremoris* subsp. *cremoris* MG1363 |  | Gasson, 1983 |
| *Lactococcus cremoris* subsp. *cremoris* MG1363 (pNZ:TuB.MgsA) | MgsA+, Cm^R^ | This work |
| *Lacticaseibacillus paracasei* BL23 |  | Mazé et al., 2010 |
| *Lacticaseibacillus paracasei* BL23 (pNZ:TuB) | Cm^R^ | This work |
| *Lacticaseibacillus paracasei* BL23 (pNZ:TuB.MgsA) | MgsA+, Cm^R^ | This work |

*Kleerebezem, M.; Boekhorst, J.; van Kranenburg, R. et al. Complete genome sequence of *Lactobacillus plantarum* WCFS1. PNAS **100** (4), 1990-1995 (2003).

*Gasson, M. J. Plasmid complements of *Streptococcus lactis* NCDO 712 and other lactic streptococci after protoplast-induced curing. J. Bacteriol. **154**, 1−9 (1983).

*Mazé, A.; Boël, G.; Zúniga, M.; Bourand, A.; Loux, V.; Yebra, M. J.; Deutscher, J.; et al. Complete genome sequence of the probiotic *Lactobacillus casei* strain BL23. J. Bacteriol. **192** (10), 2647− 2648 (2010).

Figure S1. PCR verification of the construction of *Lacticaseibacillus paracasei* BL23 (pNZ:TuB.MgsA). A *L. paracasei* BL23 colony grown in MRS agar containing chloramphenicol (5 μg/mL) was used as DNA template in a PCR reaction with oligonucleotide For-pNZ (5´-GGAATTGTCAGATAGGCCTAATGACTGG) based on the pNZ:TuB plasmid) and oligonucleotide Rv (5´ TTTTCTAGATTATAGATTGATTAGATTAGAATCTTG) based on the *mgsA* sequence from *Latilactobacillus sakei* subsp. *carnosus* DSM 15831^T^. Both oligonucleotides amplified a 729 bp DNA fragment from the pNZ:TuB.MgsA plasmid present on the *L. paracasei* BL23 strain (lane 2). A colony of *L. paracasei* BL23 (pNZ:TuB) was used as negative control (lane 3). The PCR products were subjected to horizontal agarose gel DNA electrophoresis and stained with Gel Red. A 100-bp molecular size DNA ladder (New England Biolabs) was included in the agarose gel (lane 1). Numbers indicate some of the molecular sizes. Complete gel lanes are shown.

Figure S2. Identification of ACE-βCs (AβC-COOH and AβC) in culture supernatants of *E. coli* BL21(DE3) grown in mMRS media containing glucose (2%) for 144h. HPLC-MS extracted ion chromatogram of *m/z* at 255 (M+H)^+^ (**a**) and *m/z* at 211 (M+H)^+^ (**b**) of AβC-COOH and AβC and their respective mass spectra (**c**, **d**).

Figure S3. HPLC chromatograms of culture supernatants (48 h) from *E. coli* BL21 (DE3) grown in mMRS medium containing glucose (2%) (**a**) and *L. sakei* subsp. *carnosus* DSM 15831^T^ grown in mMRS medium containing galactose (2%) (**b**) after derivatization with *o*-PDA. Detection was performed at 317 nm. Inserts: DAD spectra of the MGO derivative 2-methylquinoxaline.

Figure S4. Identification of MGO in culture supernatants of *E. coli* BL21(DE3) grown in mMRS media containing glucose (2%) for 48 h following its derivatization with *o*-PDA. HPLC-MS extracted ion chromatogram of *m/z* at 145 (M+H)^+^ (**a**), UV-Vis spectra (**b**) and mass spectra (**c**) corresponding to 2-methylquinoxaline

Figure S5. Protein alignment of methylglyoxal synthases (MgsA) from *Escherichia coli* (CAK1351959) and *Latilactobacillus sakei* subsp. *carnosus* DSM 15831^T^ (WP_056948772.1). Residues that are identical (*), conserved (:) or semiconserved (.) in both sequences are indicated. Dashes indicate gaps introduced to maximize similarities.

Figure S6. Protein alignment of putative methylglyoxal synthases (MgsA) proteins from species of the Lactobacillaceae family. The alignment showed MgsA proteins from the following type-strain: *Agrilactobacillus composti* DSM 18527^T^ (WP_035452778.1), *Agrilactobacillus fermenti* C-MHH1034^T^ (WP_230914713.1), *Lacticaseibacillus jixiensis* N163-3-2^T^ (WP_390410663.1), *Lacticaseibacillus kribbianus* YH-lac21^T^ (WP_225047161.1), *Lacticaseibacillus mingshuiensis* 117-1^T^ (WP_203626981.1), *Lacticaseibacillus nasuensis* DSM 26653^T^ (WP_056951331.1), *Lacticaseibacillus parakribbianus* YH-lacS6^T^ (WP_262314705.1), *Lacticaseibacillus saniviri* DSM 24301^T^ (KRO18005.1), *Lacticaseibacillus yichunensis* 33-1^T^ (WP_125696967.1), *Lactiplantibacillus herbarum* TCF032-E4^T^ (WP_047999447.1), *Latilactobacillus curvatus* DSM 20019^T^ (WP_004269993.1), *Latilactobacillus graminis* DSM 20719^T^ (WP_057908374.1), *Latilactobacillus fragifolli* AMBP162^T^ (WP_154241818.1), *Latilactobacillus fuchuensis* DSM14340^T^ (WP_025083183.1), *Latilactobacillus sakei* subsp. *carnosus* DSM 15831^T^ (WP_056948772.1), *Latilactobacillus sakei* subsp. *sakei* DSM 20017^T^ (WP_011374855.1), *Ligilactobacillus acidipiscis* DSM 15353^T^ (WP_010495548.1), *Ligilactobacillus pobuzihii* DSM 28122^T^ (WP_017867647.1), *Ligilactobacillus salitolerans* DSM 103433^T^ (WP_124974693.1), *Liquorilactobacillus sicerae* CUPV261^T^ (WP_281165630.1), *Liquorilactobacillus vini* DSM 20605^T^ (WP_01057989.1), *Loigolactobacillus bifermentans* DSM 20003^T^ (WP_057904775.1), *Loigolactobacillus binensis* 735-2^T^ (WP_137638180.1), *Loigolactobacillus coryniformis* subsp. *coryniformis* DSM 20001^T^ (WP_003677980.1) *Loigolactobacillus coryniformis* subsp. *torquens* DSM 20004^T^ (WP_010013276.1), *Loigolactobacillus jiayinensis* 257-1^T^ (WP_125551858.1), *Loigolactobacillus rennini* DSM 20253^T^ (WP_057873700.1), *Loigolactobacillus zhaouyuanensis* 187-3^T^ (WP_125549207.1), *Schleiferilactobacillus harbinensis* DSM 16991^T^ (WP_027828140.1), *Schleiferilactobacillus perolens* DSM 12744^T^ (WP_057822178.1), and *Schleiferilactobacillus shenzhenensis* LY-73^T^ (WP_022529735.1), *Agrilactobacillus yilanensis* 54-2 (WP_125715879.1). Residues that are identical (*), conserved (:) or semiconserved (.) in all sequences are indicated. Dashes indicate gaps introduced to maximize similarities.

Figure S7. HPLC chromatograms of culture supernatants from *L*. *paracasei* BL23-MgsA grown in mMRS medium containing glucose (2%) showing the presence of ACE-βCs at 144 h incubation (detection performed at 280 nm) (**a**) or MGO at 48 h incubation after derivatization with *o*-PDA to form 2-methylquinoxaline (detection performed at 317 nm) (**b**).

Figure S8. Bacterial growth (OD_600nm_) and presence of MGO (μM) in culture supernatants from *E. coli* BL21(DE3) grown for 48 in mMRS media containing glucose (2%) and increasing concentrations of dipotassium phosphate (0-20 g/L). MGO is represented by squares, and bacterial growth by triangles. (*) Values are significantly different (p<0.001) from the absence of phosphate added, for this and higher concentrations. The values of pH for 0, 2. 4, 8 and 20 g/L dipotassium phosphate were 6.2, 6.6, 6.8, 7.1, 7.5 (before fermentation) and 5.2, 5.2, 4.9, 5.1 and 5.0 (48 h fermentation), respectively.

Figure S9. *E. coli* BL21(DE3) growth (OD_600nm_) in mMRS media containing increasing concentrations of glucose (0-20%) with dipostassium phosphate added (2 g/L) and in the same media without phosphate added. Measures were taken at 24, 48 and 72 h of incubation. Results are mean±SEM from triplicates.

Figure S10. *E. coli* BL21(DE3) growth (OD_600nm_) in mMRS media containing glucose (2%) or in mMRS media containing glucose (2%) and dipotassium phosphate (2 g/L) in anaerobic as well as in aerobic (high oxygen/agitation) conditions. OD_600nm_ determinations were taken at 48 and 72 h of incubation. (*) Differences between aerobiosis and anaerobiosis are significant (Student´s t-test, p<0.05). Results are mean±SEM from duplicate experiments.
